# Expression of Concern: Increased Age-Adjusted Cancer Mortality After the Third mRNA-Lipid Nanoparticle Vaccine Dose During the COVID-19 Pandemic in Japan

**DOI:** 10.7759/cureus.x58

**Published:** 2024-06-12

**Authors:** Miki Gibo, Seiji Kojima, Akinori Fujisawa, Takayuki Kikuchi, Masanori Fukushima

**Affiliations:** 1 Primary Health Care, Matsubara Clinic, Kochi, JPN; 2 Pediatrics, Nagoya Pediatric Cancer Fund, Nagoya, JPN; 3 Cardiovascular Medicine, Honbetsu Cardiovascular Medicine Clinic, Honbetsu, JPN; 4 Translational Research & Health Data Science, Learning Health Society Institute, Nagoya, JPN

The Editors-in-Chief have been made aware of several concerns regarding the scientific credibility of this article. A comprehensive post-publication editorial review is being conducted to determine if any action is required.

